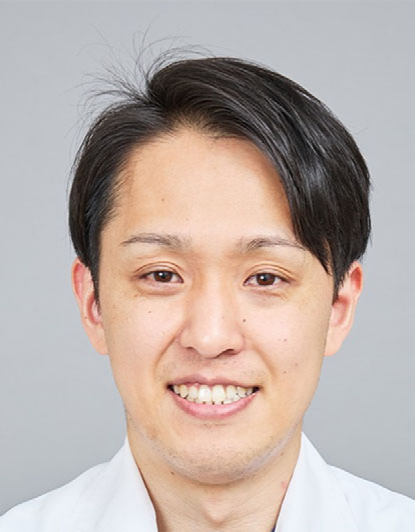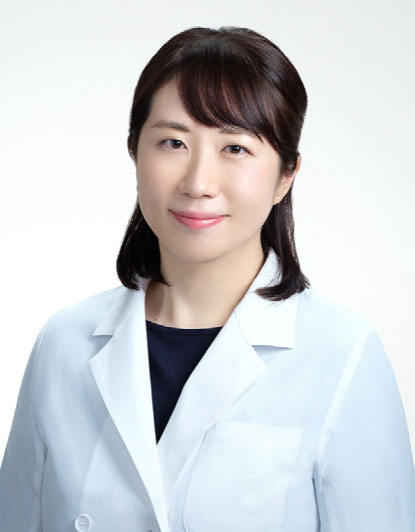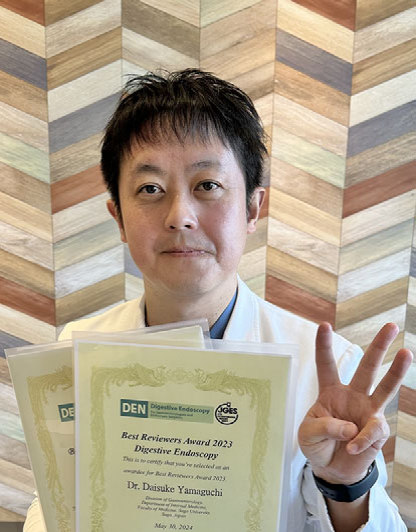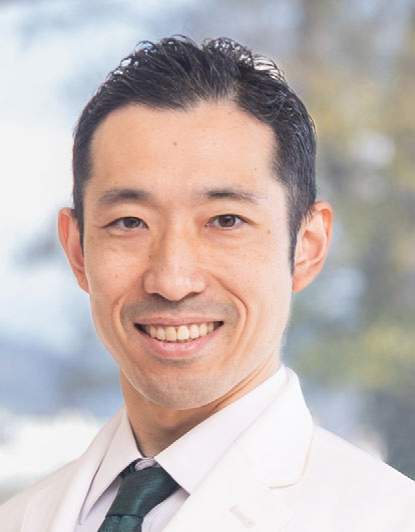# Best Reviewers Award 2025

**DOI:** 10.1002/deo2.70324

**Published:** 2026-04-15

The DEN Open Best Reviewers Award is an annual prize that recognizes the very best reviewers for their high‐quality reviews and dedication. Over 355 scholars served as reviewers in 2025, and we are pleased to announce 20 winners who have been selected based on the following criteria:

1. Invitation acceptance rate: 80% and over;
2. The number of completed reviews calculated based on;
  - Review/Original Article/Techniques and Innovation ——— × 1
  - Case Report ——— × 0.5
3. Review quality scored for each review based on; 5, Excellent/4, Good/3, Average/2, Below average/1, Poor.
4. Top 20 or more reviewers to be awarded based on the criteria below;

Total number of reviews (for 2025: 1.5 or above).
Total score of review quality.

Review period: from 1 January 2025 to 31 December 2025

**Table:** 

**Hiroko Abe**	**Mitsuru Esaki**	**Ryoji Ichijima**	**Taro Iwatsubo**
Division of Gastroenterology, Tohoku University Graduate School of Medicine, Miyagi, Japan	Division of Gastroenterology and Hepatology, Mayo Clinic Arizona, AZ, USA	Endoscopy Division, National Cancer Center, Tokyo, Japan	Second Department of Internal Medicine, Osaka Medical and Pharmaceutical University, Osaka, Japan
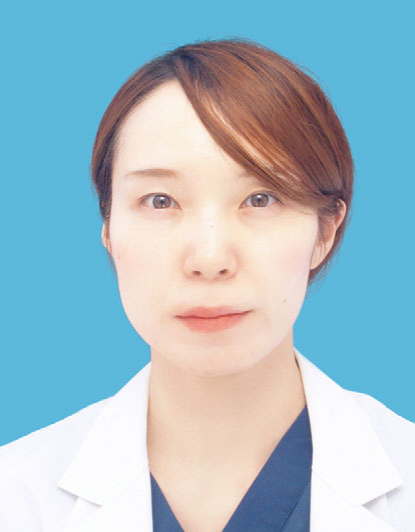	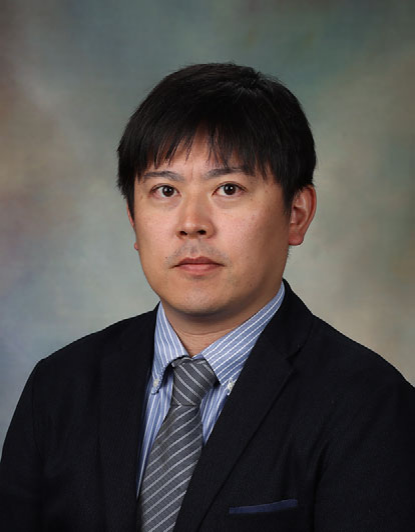	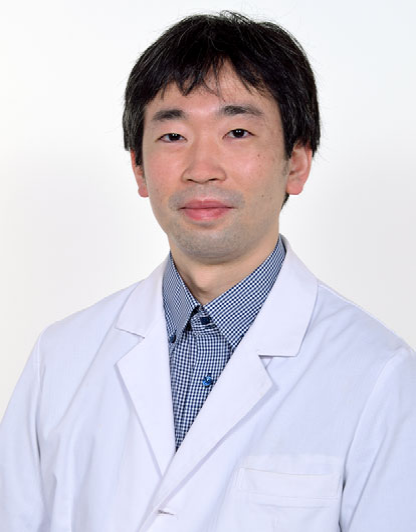	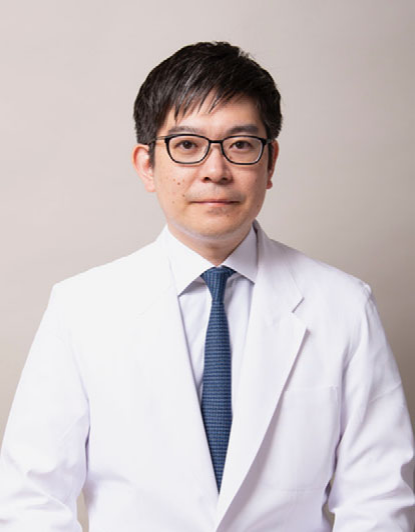
**Tsunetaka Kato**	**Koh Kitagawa**	**Hiroki Kurumi**	**Tomoaki Matsumura**
Department of Endoscopy, Fukushima Medical University Hospital, Fukushima, Japan	Department of Gastroenterology, Nara Medical University, Nara, Japan	Division of Gastroenterology and Nephrology, Department of Multidisciplinary Internal Medicine, Tottori University Faculty of Medicine, Tottori, Japan	Department of Gastroenterology, Graduate School of Medicine, Chiba University, Chiba, Japan
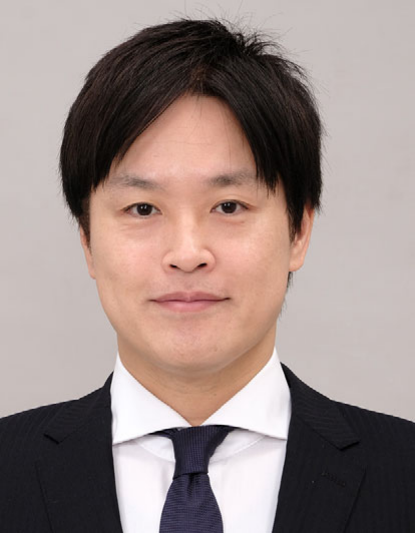	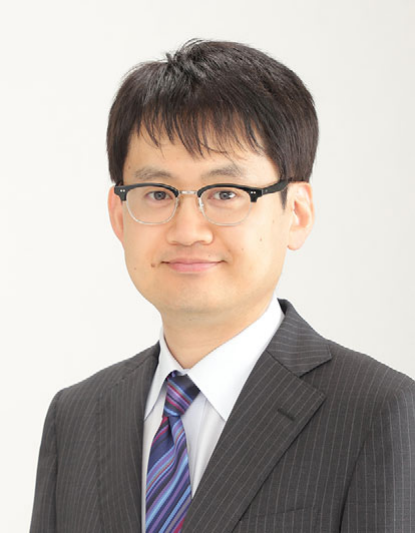	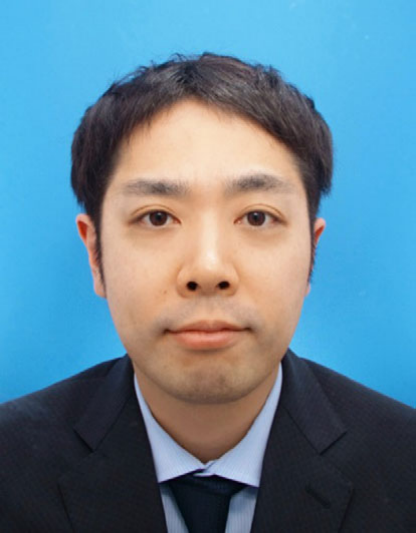	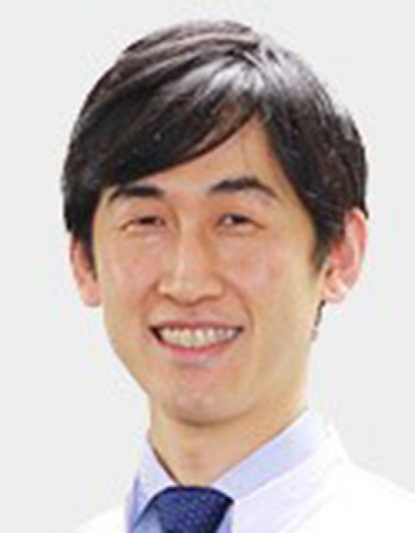
**Noriko Matsuura**	**Yohei Minato**	**Jun Nakamura**	**Ritsuko Oishi**
Division of Research and Development for Minimally Invasive Treatment, Cancer Center, Keio University School of Medicine, Tokyo, Japan	Department of Gastrointestinal Endoscopy, NTT Medical Center Tokyo, Tokyo, Japan	Department of Endoscopy, Fukushima Medical University Hospital, Fukushima, Japan	Gastroenterological Center, Yokohama City University Medical Center, Kanagawa, Japan
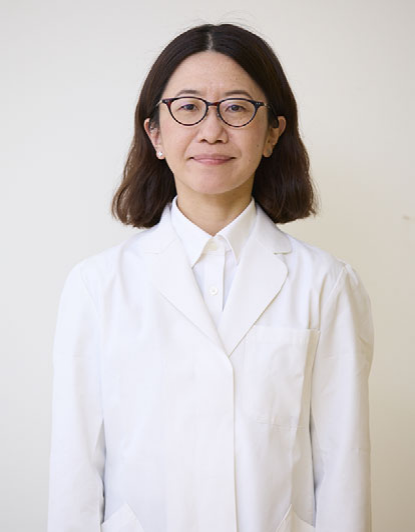	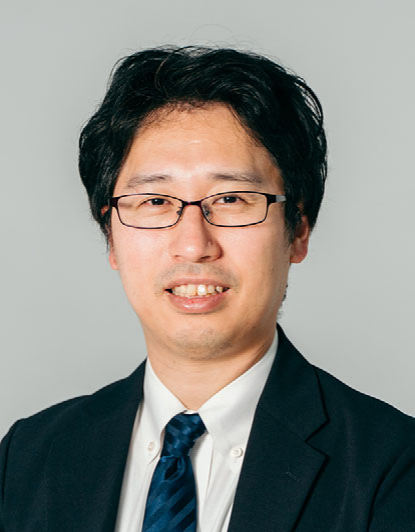	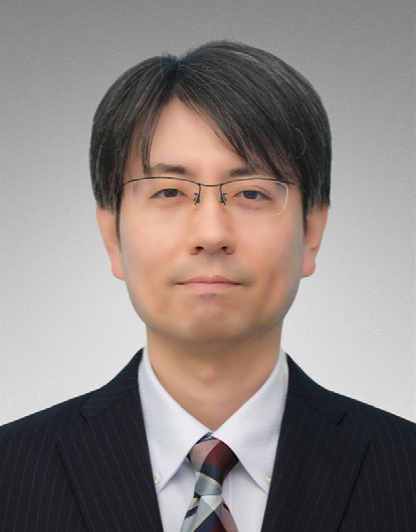	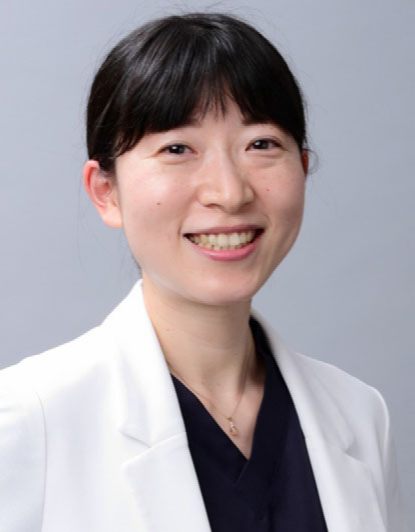
**Takeshi Okamoto**	**Taishi Okumura**	**Fumisato Sasaki**	**Noriaki Sugawara**
Department of Hepato‐Biliary‐Pancreatic Medicine, Cancer Institute Hospital of Japanese Foundation for Cancer Research, Tokyo, Japan	Digestive Disease Center, Showa Medical University Northern Yokohama Hospital, Kanagawa, Japan	Digestive and Lifestyle Diseases, Kagoshima University Graduate School of Medical and Dental Sciences, Kagoshima, Japan	Second Department of Internal Medicine (Gastroenterology), Osaka Medical and Pharmaceutical University, Osaka, Japan
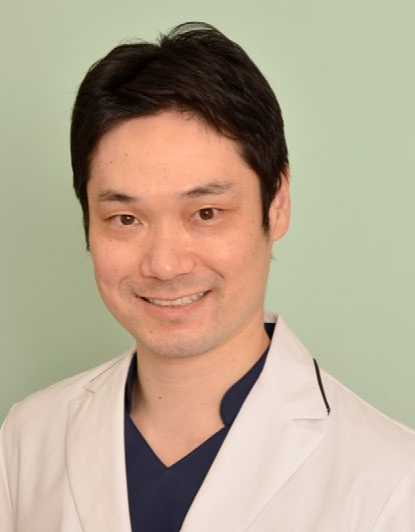	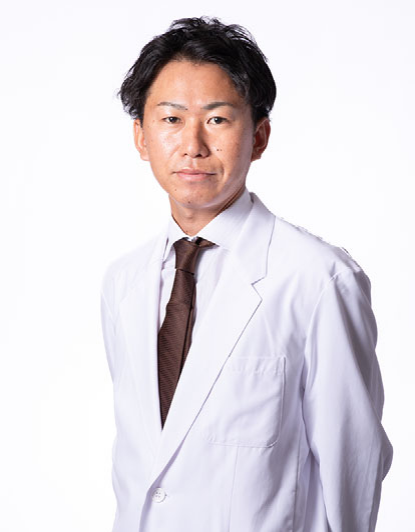	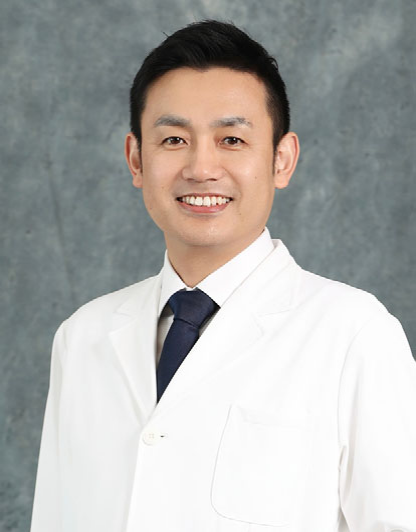	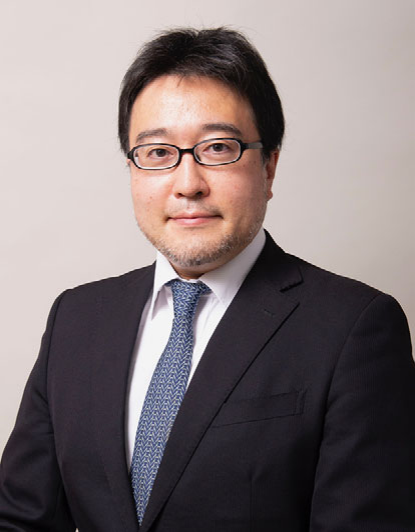
**Naoya Tada**	**Ayaka Takasu**	**Daisuke Yamaguchi**	**Takeshi Yasuda**
Division of Endoscopy, The Jikei University School of Medicine, Tokyo, Japan	Department of Gastroenterology, Cancer Institute Hospital of the Japanese Foundation for Cancer Research, Tokyo, Japan	Division of Gastroenterology, Department of Internal Medicine, Faculty of Medicine, Saga University, Saga, Japan	Molecular Gastroenterology and Hepatology, Kyoto Prefectural University of Medicine, Kyoto, Japan